# Climate change and the decline of a once common bird

**DOI:** 10.1002/ece3.95

**Published:** 2012-02

**Authors:** Christopher J W McClure, Brian W Rolek, Kenneth McDonald, Geoffrey E Hill

**Affiliations:** 1 Department of Biological Sciences, 331 Funchess Hall, Auburn UniversityAlabama 36849; 2 Archbold Biological StationAvon Park, Florida 33825

**Keywords:** Boreal forest, *Euphagus carolinus*, geographical range, global warming, *Odonata*, Pacific Decadal Oscillation, population declines, Rusty Blackbird

## Abstract

Climate change is predicted to negatively impact wildlife through a variety of mechanisms including retraction of range. We used data from the North American Breeding Bird Survey and regional and global climate indices to examine the effects of climate change on the breeding distribution of the Rusty Blackbird (*Euphagus carolinus*), a formerly common species that is rapidly declining. We found that the range of the Rusty Blackbird retracted northward by 143 km since the 1960s and that the probability of local extinction was highest at the southern range margin. Furthermore, we found that the mean breeding latitude of the Rusty Blackbird was significant and positively correlated with the Pacific Decadal Oscillation with a lag of six years. Because the annual distribution of the Rusty Blackbird is affected by annual weather patterns produced by the Pacific Decadal Oscillation, our results support the hypothesis that directional climate change over the past 40 years is contributing to the decline of the Rusty Blackbird. Our study is the first to implicate climate change, acting through range retraction, in a major decline of a formerly common bird species.

## Introduction

Climate and weather can have large effects on the life histories and distributions of animals (reviewed in:Walther et al. 2002;Sæther et al. 2004;White 2008), but documentation of such consequences requires detailed baseline information on the species in question. Birds are among the best-monitored class of vertebrates, with both well-delineated ranges and annual surveys of abundances available for many species. Such detailed knowledge of range-wide abundance makes bird species particularly suited for investigations of the effects of weather and climate on breeding biology, population trends, and distribution (Walther et al. 2002). With growing concerns about the effects of global climate change on wildlife, birds present a model taxon for monitoring the impacts of changing climate on wildlife and for exploring the mechanisms of such impacts.

Large-scale fluctuations in climate can affect local weather conditions, which in turn can impact bird populations (Sæther et al. 2004;Gemmill 2005;White 2008) either directly, by causing mortality of adults or fledglings (Newton 1998), or indirectly through bottom-up processes that change the abundance or availability of food (White 2008). When climate change makes some regions unsuitable for a particular species, then the result is a change in distribution. If there is no corresponding expansion into previously unsuitable habitat, then the resulting range retraction will result in a population decline (Thomas et al. 2006).

Among the best measures of changes in global climate patterns are the oceanic oscillations, which are deviations from average oceanic temperatures. In North America, oceanic oscillations that have been linked to changes in the breeding success and distribution of landbirds include the El Niño Southern Oscillation (ENSO, e.g.,Sillett et al. 2000), North Atlantic Oscillation (NAO, e.g.,Nott et al. 2002;Anders and Post 2006), and the Pacific Decadal Oscillation (PDO,Mantua and Hare 2002). Oscillations in the Pacific Ocean serve as indices that summarize very large scale climate patterns associated with sea surface temperatures over the southern and central Pacific, which often have a great impact on heat and precipitation load transfers over North America. The PDO describes a pattern of oceanic temperature variation over 20- to 30-year periods, and these long-term fluctuations in ocean temperature affect the climate across much of the northern portion of North America. The PDO has been shown to affect plant phenology and spring flooding in western North America (Cayan et al. 2001) as well as biomass and community structure of marine ecosystems along the Pacific coast of North America (Hare and Mantua 2000). Landbirds also are affected;Ballard et al. (2003) found that capture rates of passerines at a site in California were correlated with the PDO. Because the PDO affects both insect abundance (Kiffney et al. 2002;Vandenbosch 2003;Thomson 2009) and the timing of spring events (Cayan et al. 2001), the PDO may especially affect migratory or insectivorous bird species. Fluctuations in songbird abundances in North America may therefore best be understood within the context of the PDO (Ballard et al. 2003).

Understanding how weather and climate affect the distributions of animals with declining populations is a pressing conservation need in the face of global climate change. The Rusty Blackbird (*Euphagus carolensis*), a North American songbird that migrates from the boreal forest to southern temperate areas of the United States, has experienced persistent and severe population declines in recent decades (Greenberg and Droege 1999). Although causes of the declines remain uncertain, climate change has been hypothesized to be a contributing factor (Greenberg and Droege 1999;Greenberg and Matsuoka 2010).

The mechanism by which directional climate change could impact Rusty Blackbirds remains entirely speculative, but a study of the breeding distribution of Rusty Blackbirds in Maine documented a major range retraction within that state (Powell 2008). Range retractions in response to climate change have most frequently been implicated in the declines of relatively rare species with already small ranges such as montane frogs and birds (Thomas et al. 2006;La Sorte and Jetz 2010), The Rusty Blackbird, however, was once a common species and has a breeding distribution that stretches across northern North America (Greenberg and Droege 1999). Because retraction of range may be a common mechanism by which global climate change negatively impacts populations of animals, it is important to test for evidence of range retraction in species with declining populations.

If climate is contributing to the decline of the Rusty Blackbird, then we predicted that (1) annual fluctuations in breeding distribution should track annual fluctuations in weather, (2) local breeding populations at the southern margin of the breeding range should be more likely to go extinct compared to breeding populations away from the southern margin, and (3) over decades-long periods, the breeding distribution of Rusty Blackbirds should shift north as warming southern habitat becomes unsuitable. To test these predictions, we use data from the North American Breeding Bird Survey (BBS;Sauer et al. 2008) to examine whether the southern range boundary of the Rusty Blackbird has shifted northward and whether the probability of extinction of Rusty Blackbird populations along BBS routes decreases with latitude. Further, to determine how the mean breeding latitude of the Rusty Blackbird fluctuates with weather and climate, we examined fluctuations in the NAO, PDO, and ENSO, as well as breeding and winter temperatures in the United States and Canada. We also considered delayed effects of climate fluctuations to determine the temporal scale at which the population responds to climate.

## Material and Methods

### Study system

**Figure 1:**
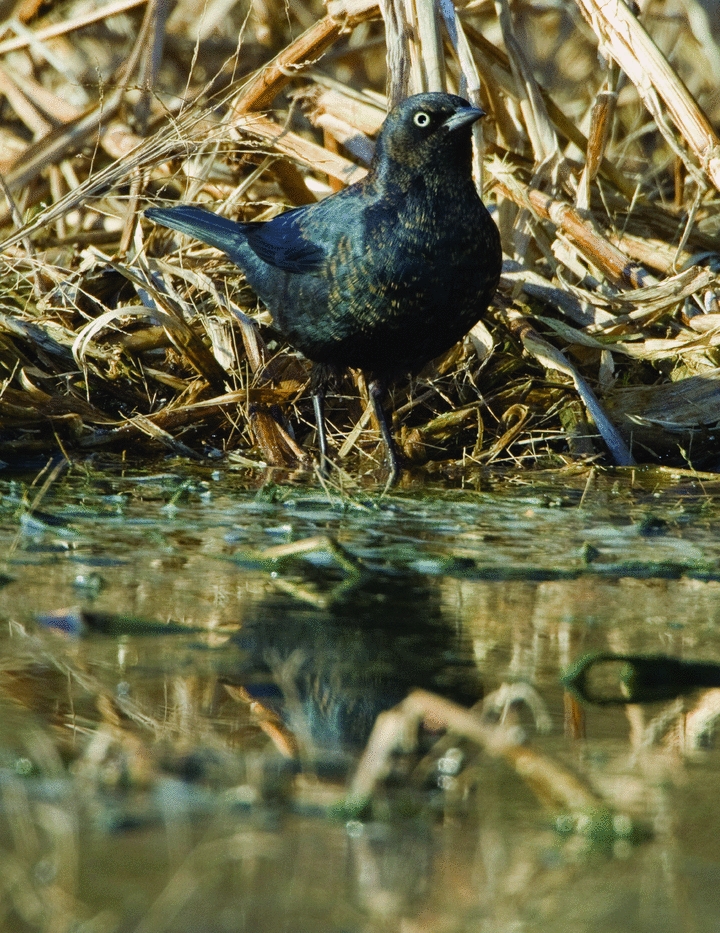
The Rusty Blackbird (*Euphagus carolinus*). Photograph: Dr. Geoffrey Hill.

The Rusty Blackbird (Fig. 1) breeds in boreal forests from New England to Alaska and north to the tree line and migrates to the southeastern United States during the winter (Avery 1995). Within the boreal forest, the Rusty Blackbird breeds in bogs, beaver (*Castor canadensis*) ponds, streamsides, and other wet forest types where it forages primarily for aquatic insects (Avery 1995;Matsuoka et al. 2010). The Rusty Blackbird also prefers wet wooded areas within its winter range, although it will also forage in agricultural areas (Avery 1995;Luscier et al. 2010). During winter, the Rusty Blackbird will eat insects as well as pine and acorn mast and some fruit (Avery 1995)

Although the Rusty Blackbird was once abundant, populations have fallen sharply over the past century (Greenberg and Droege 1999). Some authors estimate a range-wide decline of 85–95% since 1966 (Greenberg and Droege 1999; Niven et al. 2004;Sauer et al. 2008).Greenberg and Droege (1999) used qualitative data to show that populations were likely declining before monitoring programs were established. These declines have led the International Union for Conservation of Nature to designate the Rusty Blackbird as vulnerable (2009) and the U.S. Fish and Wildlife Service to list the Rusty Blackbird as a bird of conservation concern (2008).

### Bird surveys

We analyzed data from the North American BBS (Sauer et al. 2008)—a yearly, continent-wide survey of breeding birds that consists of roadside surveys conducted primarily in June. BBS volunteers drive along 35.4-km routes, stopping every 0.8 km to conduct 3-min bird counts. During each count, every bird detected within a 0.4-km radius is recorded. We calculated the average latitude at which Rusty Blackbirds were detected during each year from 1966 to 2005.

### Weather and climate variables

Weather observation data for United States and Canada were accessed from the Global Summary of the Day, National Climatic Data Center (http://www.ncdc.noaa.gov) and the Canadian Centre for Climate Modeling and Analysis (http://www.cccma.ec.gc.ca). From the National Climatic Data Center and Canadian Centre datasets, we used meteorological observations from 83 Canadian weather stations and all U.S. Stations that operated continuously throughout Rusty Blackbird wintering range during the period of observation to calculate annual mean daily temperature by hydrologic year and season. In the northern hemisphere, the hydrologic year extends from 1 October to 30 September and represents the annual hydrologic cycle—beginning when wetlands are accumulating water via precipitation, and ending when wetlands are losing water via evapotranspiration.

Data for monthly climate indices for PDO and ENSO precipitation index were obtained from the National Oceanic and Atmospheric Administration's Earth System Research Laboratory, Climate Indices/Monthly Atmospheric and Ocean Time Series Page (http://www.esrl.noaa.gov/psd/data/climateindices/list/). NAO data were obtained and downloaded from the Climate Research Unit, School of Environmental Sciences, University of East Anglia (http://www.cru.uea.ac.uk/cru/data/).

All weather data were used to calculate annual seasonal (breeding versus nonbreeding) and net means of temperature, while climate indices were used to calculate average monthly values for each year.

### Statistical analysis

#### Southern distribution shift

To test whether the entire southern range margin of the Rusty Blackbird has shifted northward over the course of the BBS, we examined the southern distribution of Rusty Blackbirds during two periods, 1967–1977 and 1998–2008, following the methods of Hitch and Leberg (2007). We analyzed the 10 most southerly routes at which Rusty Blackbirds were detected during the two focal periods using unpaired two-tailed *t*-tests. Only routes surveyed during both periods were included, which allowed us to control for changes in survey effort. By pooling data within each focal period, we were able to obtain larger sample sizes and increase the probability that Rusty Blackbirds would be detected if present. We performed analysis within two regions: one region included North American BBS routes east of –100° longitude (eastern region) and the other region included areas west of at –100° longitude (western region) to the eastern Alaskan border.

#### Local extinction

We tested the probability of local extinction for associations with latitude using a generalized linear model with a binomial distribution. We used data from elevation maps and the survey-wide BBS dataset for Rusty Blackbirds, excluding the state of Alaska, because we could not find appropriate elevation maps. We selected sites that were surveyed at least once during both periods of 1967–1977 and 1998–2008 at which Rusty Blackbirds were present during the earlier focal period. For each BBS route, we determined latitude, maximum elevation, and whether Rusty Blackbirds were detected during the later period for each individual route. Because more routes were surveyed during the interval 1998–2008 (30,850 surveys) than between 1967 and 1977 (15,803 surveys), Rusty Blackbirds should have been more likely to have been detected during the later focal decade. Thus, estimates of extinction probabilities from this analysis should yield conservative estimates. We used ESRI ArcMap 9.2 (ESRI 2008) to create a 403-m (0.25 mi) buffer around each BBS route and estimated maximum elevation for each site using the USGS national elevation dataset (Gesch 2007). Elevation data were square root transformed while latitude and longitude were scaled to zero by subtracting the minimum values. We tested whether latitude was an important predictor of the probability of a local population becoming extinct. We included elevation, longitude, and the number of surveys that occurred between 1998 and 2008 (survey effort) for each route in several models to correct for potentially confounding effects. We selected the most parsimonious model using Akaike's information criterion corrected for small sample size (AIC*_c_*, Burnham and Anderson 2002). Because spatial autocorrelation may increase type I errors, we used a Moran's I plot to test for spatial autocorrelation among residuals (Legendre and Legendre 1998).

#### Climatic fluctuations

We usedBox and Jenkins (1970) time-series analysis, autoregressive moving average (ARMA) models, to examine correlations between climatic variables and mean Rusty Blackbird latitude. To control for temporal autocorrelation, we visually assessed which ARMA models might be appropriate with a partial autocorrelation function plot and directly compared parsimony of models using AIC*_c_* and used the most parsimonious model for subsequent analyses. We tested each climatic variable in a generalized linear model for long-term trends over time. If a long-term trend was found significant (α≤ 0.05), we used the standardized residuals from the model including time to detrend the data.

The BBS has expanded north since 1966 (Sauer et al. 2008). To account for this expansion, we included mean latitude of the entire BBS north of the southernmost breeding Rusty Blackbird as a covariate in each model (herein “mean survey latitude”). We built ARMA models that included each of our measured climate variables or detrended variables. For each variable, we built a separate model for each lag of zero to eight years. We chose to analyze lags of up to eight years because Rusty Blackbirds are known to have a lifespan of up to eight years and nine months (Avery 1995); therefore, we predicted that a distributional shift could lag within a similar time period. We also built a null model that only contained mean survey latitude as an independent variable. All models were ranked and compared using AIC*_c_*. And all analyses were performed using R version 9.2.2 (R Development Core Team 2008).

## Results

**Figure 2:**
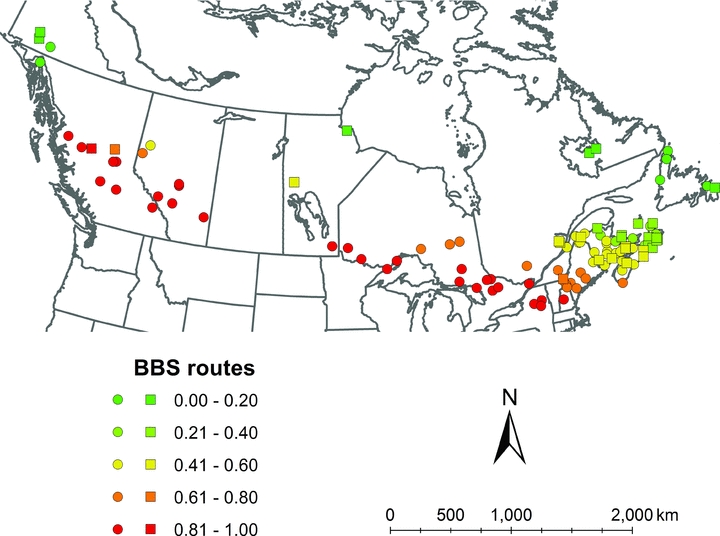
A map of the modeled probability of extinction for Rusty Blackbirds on Breeding Bird Survey (BBS) routes between the periods 1967–1977 and 1998–2008. Probability of extinction was modeled using an interaction between latitude and longitude as well as the number of times a route was surveyed 1998–2008. For mapping purposes, number of surveys was held constant at the maximum, 11. Circles represent sites at which Rusty Blackbirds were extirpated, and squares represent sites where Rusty Blackbirds persisted between the two time periods.

Rusty Blackbirds were present during 323 surveys between the years 1967 and 1977 and were present during 316 surveys from 1998 to 2008. The 100 routes at which Rusty Blackbirds were present in 1967–1977 that were surveyed again during the later decade were surveyed 659 times between 1967 and 1977 and the same routes were surveyed 670 times between 1998 and 2008. Rusty Blackbirds were locally extinct at 73 routes (73%) during the later period (Fig. 2).

Rusty Blackbird's southern range margin for the eastern region shifted 142.60 (± 40.14) km northward from 1967–1977 to 1998–2008 and this change in distribution was statistically significant (*t* = –7.06, df = 16.93, *P* < 0.0001). There were insufficient data to test the western region separately using the 10 most southerly routes where a Rusty Blackbird was detected, but a *t*-test including all western BBS routes where Rusty Blackbirds were detected during both periods showed that southern range margins in the western region also moved northward significantly (*t* = –2.56, df = 12.99, *P* = 0.02).

Moran's I test indicated nonsignificant spatial autocorrelation within the residuals of the binomial extinction variable (*P* = 0.72). The distribution-wide model for the probability of local extinction supported results from *t*-tests (Fig. 2). The model including an interaction between latitude and longitude as well as their individual effects, and survey effort received the most support and accounted for 0.79 of the AIC*_c_* weight, while the null model accounted for 0.00 of the AIC*_c_* weight (Table 1). Latitude had a negative association with the probability of extinction (β± SE) (–0.81 ± 0.22). Longitude also had a negative association with the probability of extinction (–0.15 ± 0.04), and the interaction between latitude and longitude was slightly positive (0.007 ± 0.003). As expected, survey effort had a negative association with the probability of extinction (–0.27 ± 0.10). Models that included latitude, longitude, and survey effort were well supported and accounted for 0.92 of the AIC*_c_* weight and models that included latitude accounted for all of the AIC*_c_* weight (Table 1). Overall, southern routes had a higher probability of extinction than northern routes, and models including latitude received substantial support as shown by AIC*_c_* weights (Table 1).

**Table 1:** Logistic regression models estimating the probability of local extinction for Rusty Blackbirds between the periods 1967–1977 and 1998–2008 using data from the USGS Breeding Bird Survey (BBS). Covariates used for model building included latitude, elevation, longitude, and the number of surveys conducted 1998–2008. Latitude × Longitude includes individual effects and interaction term

Models	AIC *_c_*	ΔAIC *_c_*	w _*i*_
Latitude × longitude + surveys	98.52	0	0.79
Latitude + longitude + surveys	102.11	3.59	0.13
Elevation + latitude + longitude + surveys	103.61	5.09	0.06
Elevation× latitude × longitude + surveys	106.87	8.35	0.01
Elevation + latitude + surveys	113.94	15.42	0
Elevation× latitude + surveys	116.07	17.55	0
Elevation+ surveys	116.4	17.88	0
Latitude + surveys	117.38	18.86	0
Surveys	117.39	18.87	0
Longitude + surveys	118.17	19.65	0
Null	118.78	20.26	0

**Figure 3:**
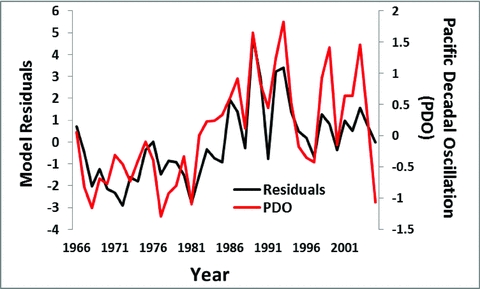
Residuals from an autoregressive moving average (ARMA) model with the mean latitude at which Rusty Blackbirds were observed on BBS routes 1966–2005 as the dependant variable plotted against the independent variable: the average latitude of all BBS Routes within the breeding range of the Rusty Blackbird as the independent variable, and the values of the Pacific Decadal Oscillation (PDO) six years prior.

Means and standard deviations of climatic variables are presented inTable 2. All independent variables except ENSO and NAO showed significant trends over time (Table 2) and were thus detrended before analysis. The average latitude of the entire BBS was positively correlated with mean Rusty Blackbird latitude (β = 3.55, SE = 0.43, df = 37, *t* = 8.25, *P* < 0.0001). While controlling for the average latitude of the BBS effort, the most parsimonious model for annual changes in the distribution of Rusty Blackbirds included PDO with a six-year lag and received 99% of AIC*_c_* weight, with the second best model being poorly supported by the data, having a ΔAIC*_c_* of 13.64 (Table 3). At a lag of six years, the PDO is positively correlated with the mean latitude of BBS routes at which the Rusty Blackbird was observed (Fig. 3, β = 1.26, SE = 0.24, df = 37, *t* = 5.27, *P* < 0.0001).

**Table 2:** Mean and standard deviation (SD) of variables examined from 1958 to 2005 as well as correlations with year (*r)* and the *P* -value of Pearson's correlation tests of correlations between a given variable and year

Climatic Variable	Mean (SD)	*r*	*P*
Average year round Canadian temperature	1.36 (0.75)	0.45	<0.01
Average breeding Canadian temperature	15.31 (0.55)	0.38	<0.01
Average winter Canadian temperature	–13.65 (1.78)	0.30	0.04
Average winter U.S. temperature	14.88 (0.53)	0.31	0.04
El Niño Southern Oscillation	0.15 (0.75)	0.27	0.06
North Atlantic Oscillation	0.04 (0.47)	–0.05	0.74
Pacific Decadal Oscillation	0.07 (0.78)	0.40	<0.01

**Table 3:** Akaike's information criterion value corrected for small sample size (AIC *_c_*), the difference in AIC *_c_* between a given model and the model with the lowest AIC *_c_* value (ΔAIC *_c_*), Akaike weights (w *_i_*) of time-series models built using the mean latitude of Rusty Blackbirds encountered on BBS routes as the dependant variable, and the independent variables listed in the first column at lags from zero to eight years. Only models receiving AICc values less that the null model are shown. A correction for shifts in survey effort was included in each model. Data were gathered from the North American BBS

Variable	Lag (years)	AIC *_c_*	ΔAIC *_c_*	w *_i_*
Pacific Decadal Oscillation	6	123.79	0	0.99
Canadian breeding temperature	7	137.43	13.64	0
El Niño Southern Oscillation	6	138.35	14.56	0
U.S. winter temperature	6	140.62	16.83	0
Annual Canadian temperature	6	141.73	17.94	0
Pacific Decadal Oscillation	4	142.60	18.81	0
North Atlantic Oscillation	0	142.63	18.84	0
Null	0	142.67	18.88	0

## Discussion

We found evidence that global climate change is negatively affecting Rusty Blackbirds by causing a retraction at the southern edge of the species’ range. We found that the southern range boundary of the Rusty Blackbird has shifted northward by an average of about 143 km since 1966. Furthermore, we found significant correspondence between annual changes in the distribution of Rusty Blackbirds and annual climatic fluctuations originating from the Pacific Ocean—annual rises in PDO corresponded with annual northward shifts in the breeding range of the Rusty Blackbird. Our observation that annual changes in the mean breeding latitude of the Rusty Blackbird is correlated with annual fluctuations in PDO suggests that long-term, directional climate change may be playing a significant role in the decline of the Rusty Blackbird. Our data do not discount alternative explanations for the decline of the Rusty Blackbird such as mercury poisoning (Edmonds et al. 2010), disease (Barnard et al. 2010) and habitat destruction (Powell et al. 2010). However, the correlation between the continent-wide distribution of the Rusty Blackbird and climatic fluctuations suggests that directional climate change is a major factor in observed declines of Rusty Blackbird populations. To our knowledge, this is the first study to show that global climate changes can negatively impact a common bird species by causing a retraction of its range.

Declining species often show range retractions even when climate change is not a factor (Wilcove and Terborgh 1984), so it is possible that the range retraction of Rusty Blackbirds and rising temperatures are not causally linked. However, range retraction simply due to declining populations should present different patterns of distribution change compared to range retraction due to climate change. If range retraction is due to increasing scarcity of a species, then loss of range should occur along the entire periphery of the species’ range. In contrast, under range retraction due to climate change, we expect low-latitude populations to decline, while high-latitude populations remain stable. Our study confirms suggestions by previous researchers (Machtans et al. 2007, B. W. Rolek et al. unpubl. data) that Rusty Blackbird populations are declining disproportionately at low-latitude range margins. Because the treeline is moving northward due to a warming arctic climate (Holtmeier and Broll 2005), it is also possible that the northern range of the Rusty Blackbird is expanding northward (Norment et al. 1999). We could not address expansion at the northern margin because the BBS does not cover the northern range boundary of the Rusty Blackbird. Future monitoring efforts should assess to what extent northward expansion may offset retraction at the southern margins.

By what mechanism can the surface temperature of the Pacific Ocean be linked to the success of a songbird breeding in wetlands in the boreal forest? The breeding success and site selection of Rusty Blackbirds is closely tied to shallow water and macroinverterbrate prey, particularly odonates (Matsuoka et al. 2010). The observed changes in the breeding range of the Rusty Blackbird may be driven, at least in part, by rising annual temperatures depressing the abundance of macroinverterbrate prey that breed in wetlands. Changes in rainfall and temperatures affect moisture in bogs, which affects odonates and hence blackbirds.

Negative effects of rising global temperatures might also extend beyond southern range boundaries as warmer temperatures decrease the abundance and size of stream invertebrates within the breeding range of the Rusty Blackbird (Hogg and Williams 1996). Increasing temperatures can also alter invertebrate community structure of lakes within boreal forest (Flenner and Sahlén 2008). In Alaska, climate change is causing wetlands to dry and is causing shifts from a community dominated by macroinvertebrates to a community consisting mostly of zooplankton (Corcoran et al. 2009). The PDO is negatively correlated with amount of organic matter in streams, thus affecting the macroinvertebrate community and vertebrates that depend on it (Kiffney et al. 2002).

Besides a reduction in odonate numbers, climate change may also affect the phenology of odonates, shifting them out of phase from the phenology of breeding Rusty Blackbirds. In Britain, odonate emergence has been observed to shift three to seven days earlier per one degree increase in temperature (Hassall et al. 2007). Canadian stream invertebrates have also been shown to emerge earlier in response to warmer temperatures (Hogg and Williams 1996). A shift in phenology of invertebrates has been shown to negatively affect bird species, and particularly migratory bird species, that feed upon them (Thomas et al. 2001;Both et al. 2006;Møller et al. 2008). As a migratory species, the Rusty Blackbird may be especially influenced by the changing phenology of its prey due to the PDO. Species of birds associated with boreal wetlands are declining across North America (Sauer et al. 2008), and in Finland, migratory birds and birds associated with wetlands have shown pronounced shifts in range (Brommer 2008). The effects of global warming on lower trophic levels may therefore affect entire wetland communities, highlighting the need to examine the effects of climate change on widespread species.

Shifting prey phenology may also explain the six-year lag that we observed in the shift in mean breeding latitude of Rusty Blackbirds in response to the PDO. Birds often lag in their response to changes in phenology of species at lower trophic levels (Visser et al. 2004;Both et al. 2009) because of constraints on phenotypic plasticity (Both and Visser 2001,Visser et al. 2004). For instance, migratory species may lag in response to shifting prey abundance if timing of their migration is determined by factors unrelated to climatic conditions (Both and Visser 2001,Visser et al. 2004). The Rusty Blackbird may therefore be especially prone to such lags in response to changes in prey phenology because of additional constraints imposed on breeding date by the timing of migration.

Our study lends support to the hypothesis that climate change is contributing to the decline of Rusty Blackbird populations (Greenberg and Droege 1999; Greenberg and Matsuoka 2010). Our observations of Rusty Blackbirds suggest that such range shifts can be the basis for declines of even abundant and widespread species when retraction at the southern edge of a range may not be offset by range expansion at the northern edge. The Rusty Blackbird is the most widespread vertebrate for which a range shift has been implicated as a cause for population decline, but climate-induced range shifts may be an underappreciated cause for the decline of other animal and plant species (Thomas et al. 2006;La Sorte and Jetz 2010).
